# Polycystic ovary syndrome and its impact on Iranian women’s quality of life: a population-based study

**DOI:** 10.1186/s12905-018-0658-1

**Published:** 2018-10-11

**Authors:** Zahra Behboodi Moghadam, Bita Fereidooni, Mohsen Saffari, Ali Montazeri

**Affiliations:** 1 Department of Reproductive Health, School of Nursing and Midwifery, Tehran University of Medical Sciences, Tehran, Iran; 2 Health Research Center, Life Style Institute, Baqiyatallah University of Medical Sciences, Tehran, Iran; 3 Population Health Research Group, Health Metrics Research Center, Iranian Institute for Health Sciences Research, ACECR, Tehran, Iran

**Keywords:** Polycystic ovary syndrome, Health-related quality of life, Iran

## Abstract

**Background:**

Polycystic ovary syndrome (PCOS) is a major public health concern worldwide affecting up to one in five women at reproductive age. It is associated with biochemical and hormonal disturbances as well as adverse cosmetic, reproductive, metabolic, and psychological consequences, resulting in worsened quality of life. The aim of the present study is evaluating the quality of life and determining its degrading factors among Iranian women suffering from this syndrome.

**Methods:**

This cross-sectional study was conducted on 200 PCOS women in Hamadan, Iran. In order to measure quality of life we used the Persian version of Health-related Quality of Life Questionnaire for PCOS (PCOSQ). Descriptive statistics was used to explore the data. In addition linear regression analysis was performed to assess factors affecting health-related quality of life in this population.

**Results:**

The mean score for quality of life domains (from the greatest to the least serious concern) were: infertility (3.43 ± 1.63), emotions (3.55 ± 1.17), menstrual problems (3.77 ± 1.36), body hair (3.80 ± 2.05) and weight (4.32 ± 1.80), respectively. The higher score represents better function. However, multivariate analysis revealed that hirsutism had the strongest impact on the patients’ quality of life (*p* < 0.001) followed by infertility (*p* = 0.038) and menstrual irregularity (*p* = 0.003).

**Conclusion:**

The findings showed that impairment of quality of life was associated with PCOS related conditions such as hirsutism**,** infertility and menstrual problems.

## Background

Polycystic ovary syndrome (PCOS) is a heterogeneous, multifactorial disorder that affects 12–21% of women population at reproductive age depending on the diagnostic criteria and ethnicity [1, 2]. The prevalence of the syndrome in Iran has been reported to be between 7.1 and 14.6% [3]. The pathophysiology of PCOS remains unclear, but it is believed that it results from complex interactions between genetic, metabolic, and environmental factors [4–6]. It is characterized by hyperandrogenism, ovulatory dysfunction and polycystic ovarian morphology [7].

PCOS is the most common hormonal disturbance causing lifelong physical and financial burden [4, 8, 9] and can affect women’s health even after menopause [10]. It is considered as leading cause of infertility [11] and is associated with adverse clinical complications including reproductive (menstrual irregularity, infertility) [1, 4], metabolic (insulin resistance, diabetes mellitus, cardiovascular risk) [12], and psychological disability (anxiety, depression) [13, 14].

These threatening complications can lead to mood disorders, eating disorders, social and marital conflicts and sexual dysfunction [13, 15–18]. All have been mentioned as reasons for significant reduction in HRQoL in multiple studies and meta-analyses [19–23].

Health-related quality of life (HRQoL) is a multidimensional concept used to describe physical, emotional and social aspects of particular diseases or its treatment [24, 25]. HRQoL is widely considered as an important parameter for evaluating the quality and outcome of health care, especially in chronic disorders such as PCOS [26].

PCOS occurs in all ethnic groups with differing prevalence depending on body weight, diet, lifestyle, and ethnic background [27]. But, the importance of its dimensions varies in societies depending on the religious, racial, cultural and social factors [28, 29]. Excessive body weight has been widely reported as an important concern to women with PCOS especially in adolescents [21, 30]. Hirsutism is present in approximately 70% of women with PCOS and is often cited by patients as being one of the most disturbing aspects of PCOS [1, 31]. Women with PCOS who experience hirsutism have often expressed that they feel ‘unfeminine’ [32, 33].

Another feature of PCOS is infertility, which has been related to negative psychological symptoms [4, 30] and decreased quality of life in affected women [34]. ‘Feeling inferior, and less of a woman’, was talked about in relation to being infertile in a recent qualitative study in Iran [35].

Finally, menstrual irregularities such as amenorrhea and oligomenorrhea are common experiences among women with PCOS [12]. Semi-structured interviews with women with PCOS revealed that menstrual irregularities are associated with low feminine identity [33, 36].

Sociocultural factors may lead some women to perceive menstrual problems and infertility as more problematic than other features of PCOS [29]. Therefore, the purpose of this cross-sectional research was to assess HRQoL and to determine factors related to its impairment among Iranian women, affected by the syndrome.

## Methods

### Design and participants

This was a cross-sectional study carried out with a sample of women with PCOS who attended three clinics (gynecologic, infertility, and dermatology) in Hamadan, Iran from October 2016to March 2017. The PCOS diagnosis was confirmed by visiting gynecologists based on the Rotterdam diagnostic criteria, i.e. having any two of the followings: Oligo/amenorrhea, clinical and/or biochemical hyperandrogenism, and polycystic ovaries (presence of 12 follicles or more in one or both ovaries and/or increased ovarian volume i.e. > 10 ml) [7]. Women who had been previously diagnosed with PCOS and were scheduled for a routine follow-up appointment were invited to participate in the study. Patients were eligible if they met each of the following criteria: being 18–45 years of age, Iranian, being able to read and write, not having non-classic adrenal hyperplasia, thyroid or metabolic disease and hyperprolactinemia, not being previously diagnosed with psychiatric disorders and not using psychiatric medications including antidepressants for at least 3 months before entering the study. Single women were included in the study, as the possibility of being infertile is an important concern among adolescent girls suffering from PCOS [37, 38].

After explaining the objectives of the study, written informed consent was obtained from each participant.

### Measures

1. Self-reported basic demographic information including age, marital status, education, employment status, household income, and reproductive status. Household income was indicated based monthly salary and was categorized as: good, fair, and poor.
2. Body mass index (BMI) was determined for all participants.
3. Hirsutism was assessed using the Ferriman-Gallwey scoring method (F/G score) by a trained interviewer under the supervision of a physician. F/G score range from 0 to 36 and scores of 8 or higher are considered of having hirsutism [39, 40].
4. Menstrual history: all patients were asked about the interval between menstruation during preceding 12 months and were categorized into groups of: < 21 days, 21–34 days, 35–60 days (oligomenorrhea), > 199 days (amenorrhea) and variable. Infertility history (12 months of unprotected sexual intercourse without conception) was also recorded for each woman.
5. Health-related Quality of Life Questionnaire for PCOS (PCOSQ), which includes 26 items tapping into five HRQoL domains: emotions (8 items), weight (5 items), body hair (5 items), infertility (4 items) and menstrual problems (4 items) [41]. Patients were asked to choose the response option that best suited their feelings during the past 2 weeks. Response categories are rated on a seven-point Likert scale and scores was calculated based on the recommended procedure giving a score from 1 to 7 for each domain where the higher score represents better function. The psychometric properties of the Iranian version of the questionnaire are well documented [42, 43].

### Data analysis

Descriptive analyses were performed to provide a summary of findings. The SPSS version 16 (SPSS Inc., Chicago, IL, USA) was used for data analysis. Comparison of continuous data was performed using one-way analysis of variance (ANOVA) and t-test. Linear regression analysis (enter method) was used to assess factors associated with HRQoL while adjusting for demographic (age, education level, residency, marital status, financial status, occupation) and clinical (BMI, hirsutism score, infertility history, menstrual irregularity) variables. The normal distribution of the data was examined graphically by normal probability plots and tested by the Kolmogorov-Smirnov test. *P* values less than 0.05 were considered significant.

## Results

In all 316 women were approached. Of these 116 women did not met eligibility criteria and thus 200 patients entered into the study. Demographic and clinical characteristics of the participants are presented in Table 1. As shown, the mean age of participants was 27.9 ± 5.88 years. The majority of women were housewives (79.5%), ever married (73.5%), and with secondary education or higher (80%). The mean BMI was 26.56 ± 4.33 kg/m^2^. Overall 35.5% and of the participating women were overweight and 26.5% were obese, respectively (BMI of 25–29.9 indicates being overweight and a BMI of 30 and higher indicates obesity). Infertility was reported in 63% of the married participants and 73.5% of all cases had irregularity in their menstrual cycles. Approximately, two thirds of the women exhibited clinical features of hirsutism, with mean F/G scores of 12.88 ± 8.81.

**Table 1:** Socio-demographic and clinical characteristics of the study participants (*n* = 200)

	Number	Percent
*Demographic information*		
Age (Mean, SD)	27.9 ± 5.88	
Education		
Primary	40.0	20
Secondary	63.0	31.5
Higher	97.0	48.5
Occupation		
Housewife	159.0	79.5
Employed	33.0	16.5
Student	8.0	4
Household income		
Poor	55.0	27.5
Fair	122.0	61
Good	23.0	11.5
Marital status		
Ever married	147.0	73.5
Never married	53.0	26.5
*Clinical information*		
BMI (kg/m2)		
< 25	76.0	38
25–30	71.0	35.5
≥ 30	53.0	26.5
F/G^a^ score (Mean, SD)	12.88 ± 8.81	–
History of infertility in married patients (*n* = 147)		
Yes	93.0	63
No	54.0	37
Interval between menstruation (days)		
< 21	9.0	4.5
21–34	53.0	26.5
35–60	44.0	22
> 199	9.0	4.5
Variable	85.0	42.5

^a^ Ferriman-Gallwey score

The mean scores for the HRQoL domains obtained from the questionnaire showed that the lowest score was for infertility domain (3.43 ± 1.63 out of 7) and the highest score was for the weight domain (4.32 ± 1.80 out of 7). Younger women (18–24 years) had higher emotions and weight domain scores compared with other age groups. Household income was not associated with any of the domains of the HRQoL. Women with higher BMI had lower scores in weight domain. The detailed results for the whole sample and for sub-samples are presented in Table 2. However, after adjusting data for demographic and clinical variables, it was found that hirsutism (*p* < 0.001) followed by infertility (*p* = 0.038) and menstrual problems (*P* = 0.003) had the strongest impact on the patients’ HRQoL. Emotions score decreased with age and hirsutism score. The hirsutism score also significantly predicted reductions in all domain scores except for menstrual problems (*p* < 0.05). The findings are shown in Table 3.

**Table 2:** HRQoL scores in sub groups obtained from univariate analysis

All		Emotions	Body hair	Weight	Infertility	Menstrual problems
	N	Mean (SD)	Mean (SD)	Mean (SD)	Mean (SD)	Mean (SD)
	200	3.55 ± 1.17	3.80 ± 2.05	4.32 ± 1.80	3.43 ± 1.63	3.77 ± 1.36
Age group (year) ^b^						
18–24	60.0	3.86 ± 1.06	4.09 ± 2.02	4.69 ± 1.88	3.71 ± 1.57	4.02 ± 1.33
25–29	67.0	3.65 ± 1.21	4.13 ± 2.21	4.66 ± 1.81	3.16 ± 1.59	3.78 ± 1.31
30–34	45.0	3.18 ± 1.18	2.98 ± 1.79	3.66 ± 1.58	3.28 ± 1.62	3.51 ± 1.48
35–45	28.0	3.24 ± 1.08	3.65 ± 1.81	3.76 ± 1.58	3.69 ± 1.83	3.06 ± 1.36
p-value		0.011*	0.025*	0.003*	0.202	0.291
Marital status ^b^						
Ever married	147.0	3.72 ± 1.13	3.18 ± 1.64	4.44 ± 1.76	3.67 ± 1.48	3.84 ± 1.46
Never married	43.0	3.53 ± 1.17	4.07 ± 2.14	4.32 ± 1.82	3.30 ± 1.67	3.80 ± 1.31
*p*-value		0.291	0.007*	0.682	0.156	0.833
Residency ^b^						
Urban	150.0	3.54 ± 1.16	3.74 ± 2.03	4.21 ± 1.79	3.44 ± 1.59	3.81 ± 1.36
Rural	50.0	3.60 ± 1.20	3.96 ± 2.11	4.66 ± 1.81	3.39 ± 1.75	3.66 ± 1.37
p-value		0.629	0.683	0.857	0.341	0.557
Education level ^a^						
Primary	40.0	3.52 ± 1.14	4.45 ± 2.05	4.60 ± 1.75	3.31 ± 1.57	3.63 ± 1.26
Secondary	63.0	3.47 ± 1.03	4.14 ± 2.20	4.06 ± 1.73	3.31 ± 1.79	3.79 ± 1.29
Higher	97.0	3.62 ± 1.26	3.30 ± 1.84	4.38 ± 1.87	3.55 ± 1.55	3.82 ± 1.46
*p*-value		0.713	0.031*	0.312	0.590	0.766
Household income ^a^						
Poor	55.0	3.39 ± 1.09	3.78 ± 2.14	4.47 ± 1.67	3.23 ± 1.66	3.72 ± 1.38
Fair	122.0	3.60 ± 1.22	3.87 ± 2.06	4.30 ± 1.89	3.41 ± 1.61	3.74 ± 1.33
Good	23.0	3.72 ± 1.07	3.44 ± 1.80	4.09 ± 1.68	4.01 ± 1.62	4.09 ± 1.51
p-value		0.413	0.651	0.656	0.153	0.492
Occupation ^a^						
Housewife	159.0	3.54 ± 1.62	3.80 ± 2.07	4.24 ± 1.83	3.44 ± 1.64	3.78 ± 1.42
Employed	33.0	3.66 ± 1.14	3.92 ± 1.91	4.66 ± 1.71	3.52 ± 1.54	3.60 ± 1.07
Student	8.0	3.32 ± 1.52	3.22 ± 2.27	4.62 ± 1.55	2.87 ± 1.79	4.28 ± 1.27
p-value		0.740	0.688	0.433	0.598	0.448
BMI(kg/m2) ^a^						
< 25	76.0	3.63 ± 1.17	3.97 ± 2.11	5.45 ± 1.61	3.30 ± 1.58	3.89 ± 1.36
25–30	71.0	3.54 ± 1.21	3.83 ± 2.05	3.99 ± 1.69	3.36 ± 1.66	3.65 ± 1.34
≥ 30	53.0	3.46 ± 1.12	3.52 ± 1.99	3.17 ± 1.25	3.70 ± 1.67	3.78 ± 1.42
p-value		0.687	0.481	< 0.001*	0.361	0.579
Hirsutism ^b^						
Yes	144	3.47 ± 1.17	2.75 ± 1.33	5.03 ± 1.61	3.47 ± 1.62	3.72 ± 1.35
No	56.0	3.74 ± 1.14	6.48 ± 0.66	4.25 ± 1.80	3.32 ± 1.67	3.91 ± 1.39
p-value		0.727	< 0.001*	0.121	0.667	0.541
History of infertility ^b^						
Yes	93.0	3.53 ± 1.14	3.36 ± 1.09	4.22 ± 1.75	2.85 ± 1.39	3.86 ± 1.27
No	54.0	3.44 ± 1.25	3.40 ± 2.08	4.35 ± 1.96	4.28 ± 1.77	3.62 ± 1.40
*p*-value		0.642	< 0.001*	0.693	< 0.001*	0.297
Menstrual Irregularity ^b^						
Yes	53.0	3.40 ± 1.16	3.77 ± 2.09	4.16 ± 1.82	3.41 ± 1.66	3.52 ± 1.26
No	147.0	3.97 ± 1.09	3.86 ± 1.96	4.78 ± 1.70	4.46 ± 1.54	4.46 ± 1.41
p-value		< 0.001*	0.792	0.034	0.857	< 0.001*

^a^ derived from ANOVA

^b^ derived from t test

* *p* < 0.05

**Table 3:** Linear regression analysis of possible related factors in HRQoL and domains

	HRQoL	Emotions	Body hair	Weight	Infertility	Menstrual problems
	β	β	β	β	β	β
Age ^a^	−0.104	− 0.198*	− 0.068	− 0.027	0.085	− 0.181*
BMI ^a^	−0.104	0.060	0.026	−0.540*	0.142	0.069
Hirsutism score ^a^	−0.566*	−0.235*	−0.849*	−0.205*	−0.190*	− 0.124
Marital status ^b^						
Never married Ever married (Ref)	0.059	0.035	−0.103*	− 0.022	0.371*	− 0.074
Residency ^b^						
Rural Urban (Ref)	−0.008	0.043	−0.053	0.024	0.018	−0.040
Financial Status ^b^						
Low High (Ref)	0.028	0.070	0.034	−0.003	0.009	−0.014
Education						
Primary < Secondary and higher (Ref)	−0.087	0.001	0.079*	0.135*	− 0.092	−0.087
Occupation ^b^						
Employed Housewife (Ref)	−0.027	−0.004	− 0.044	−0.006	0.023	−0.050
Infertility ^b^						
Yes No (Ref)	−0.219*	0.008	0.040	− 0.072	−0.465*	0.089
Menstrual Irregularity ^b^						
Yes No (Ref)	−0.167*	−0.212*	0.034	−0.147*	0.009	−0.302*
Adjusted R^2^	0.39	0.11	0.77	0.38	0.15	0.10

^a^ β is reported as association amounts

^b^ β is reported as mean differences of scores between two groups

* *p* < 0.05

## Discussion

This study sought to extend prior research by determining HRQoL and related factors in PCOS women in Iran. The result of our study showed that hirsutism, had the most significant impact on the HRQol. This is consistent with findings from other studies that revealed hirsutism as a significant predictor of psychological distress [44] and HRQoL [15, 45] in PCOS women.

It is argued that physical, social and psychological consequences of PCOS have an important influence on patients’ quality of life [35]. However, severity of the problem varies according to socio-cultural factors, traditions, and religious beliefs [29, 46].

Issues with weight and hirsutism result in women reporting feeling ‘less feminine’ than the societal ideal definition for thin and hairless bodies for women [47]. Similar to the findings of other studies [5, 48], F/G score predicted emotion subscale score on the PCOSQ in our study.

Infertility was the second most important contributor to a decreased HRQoL score. It was also reported as the most common HRQoL concern by participants. As noted earlier, more than half of the married women in the study had infertility.

The diagnosis of infertility is almost always accompanied with the great psychological distress, so-called ‘infertility stress’ [49]. Motherhood has been considered as an important component of women’s identity and social role for a large group of patients with PCOS [50] and wishing to conceive, received a higher priority for them in comparison to other infertile patients [51]. However, the extent to which infertility can affect HRQoL is highly dependent on cultural, ethnic and social factors [48].

Our findings may be in line with new pronatalist policy in Iran, which started since 2012 in response to a significant decline in the fertility rate. In pronatalist societies, there are pro birth policies put in place by the government that encourage parenthood and reproduction [52]. This is added pressure on couples to reproduce and follow what society has prescribed as normal [53, 54].

Therefore, infertility may be perceived as a major concern for Iranian women due to social pressure for having children by the society. Our findings are consistent with experiences of Iranian women in a recent qualitative study which showed that PCOS-related infertility can be a major HRQoL concern [35].

Bazarganipour and her colleagues in a cross-sectional study on 300 married Iranian women with PCOS showed that infertility and menstrual domains were the most affected areas of HRQoL [55]. However, all patients in this study were married. Moreover, patients were recruited from two private gynecology clinics. Such patients might differ in a number of sociocultural backgrounds compared with a community sample. Thus, it is necessary to provide appropriate strategies for improving fertility in these patients. Educating women about potential fertility problems and informing them in order to get involved in healthy lifestyle practices are important. Providing a structured education can be also beneficial for patients’ understanding of their condition and improving their quality of life [56].

Contrary to earlier reports that body weight issues played a major role in lowering the quality of life, it was not associated with HRQoL and received the least prominence among the other subscales reported as areas of concern in our study. It probably reflects different sociocultural views of obesity symptoms [57].

In western industrialized societies, obesity is perceived as a negative and abnormal feature and fear of being overweight is much more prevalent than in developing countries [58]. So, in their most studies, it was weight, which mostly affected HRQoL. According to the results of two American and German studies, body weight domain was strongly associated with lower HRQoL [5, 59].

Jones et al. in United Kingdom found weight problems as one of the most important factors negatively affecting QoL [38]. The results of a recent Italian study showed a considerable worsening of HRQoL in obese PCOS patients compared with controls [23]. Indeed, it seems that hirsutism and infertility perhaps play a more important role in having a negative impact on HRQOL than obesity among the Iranian women.

Similarly, a study comparing Austrians and Muslim immigrants found menstrual irregularity and infertility to be as a bigger problems than overweight or obesity in Muslims, whereas European women perceived these quite differently [29].

In fact, differences in perception and body image represented in PCOS women may be conditioned by cultural influences. We also found that, menstrual irregularity decreased the QoL. Menstrual dysfunction is considered one of the hallmarks of PCOS, identified in the majority (70–80%) of patients [4]. A negative effect of menstrual problems on the HRQOL and psychological distress of patients has been previously discussed by other authors [5, 60].

To mention the strengths of our study, it is worthy to mention that this study enjoys sample diversity with various phenotypes of PCOS from different socioeconomic backgrounds which may have resulted in a comprehensive assessment. But, our participants were chosen from outpatients, and the findings of this study may not be applicable to those with milder PCOS phenotypes, who had never referred to any health centers for treatment. This can be a limitation of our study.

## Conclusion

The findings indicated that quality of life impairment in women with PCOS was associated with hirsutism, infertility and menstrual irregularity. Indeed appropriate management strategies are needed to improve quality of life in these women.

## Abbreviations

ANOVA: One-way analysis of variance
BMI: Body mass index
F/G: Ferriman-Gallwey
HRQOL: Health-related quality of life
PCOS: Polycystic ovary syndrome
PCOSQ: Polycystic ovary syndrome questionnaire
